# Beyond memory: exploring the value of social cognition for older adults with neurocognitive disorders

**DOI:** 10.3389/fpsyt.2023.1209745

**Published:** 2023-09-28

**Authors:** Suraj Samtani, Anjani Meka, Joyce Siette

**Affiliations:** ^1^Centre for Healthy Brain Ageing (CHeBA), Discipline of Psychiatry and Mental Health, Faculty of Medicine and Health, University of New South Wales, Sydney, NSW, Australia; ^2^The MARCS Institute for Brain, Behaviour and Development, Western Sydney University, Westmead, NSW, Australia

**Keywords:** social cognition, assessment, older adults, mild cognitive impairment, dementia

## Abstract

Neurocognitive disorders involves progressive decline in cognition, function, behavior and needs. Recent developments have identified the need to characterize social cognition in individuals with neurocognitive impairments to support uncertainty in clinical decision making, treatment plans and monitoring individual change. Routine social cognition assessments have thus been more recently used and adopted in persons with dementia or mild cognitive impairment. This work serves to summarize current assessments and provide a discourse on the practicality of available social cognition tools, its implication in clinical practice and key future directions. We highlight advantages in establishing validated, multicomponent measures of social cognition for people with neurocognitive disorders.

## Introduction

Humans are inherently social beings, and being socially connected with others is a basic human need (1). Yet as we age, we experience a range of physical, cognitive and social changes that can impact our daily functioning and subsequently our wellbeing. In this context, social cognition, which refers to our ability to recognize emotions, social cues, inhibit inappropriate behavior and act appropriately in social situations (2), is a key component of social functioning and can be affected by age-related changes (3). Key social cognitive domains include emotion perception (the ability to identify emotions), theory of mind (the ability to understand others’ mental states) including cognitive theory of mind (understanding others’ thoughts, beliefs or intentions), affective theory of mind (inferring others’ emotional states), empathy (mirroring another’s affective state), and social perception (understanding social cues) (4, 5). Empathy has a cognitive (understanding others’ emotions) and an affective component (feeling what others feel), of which the former overlaps with affective theory of mind, but also includes emotional contagion (unintentional mimicry and synchronizing with others’ emotions) (6, 7).

Social cognitive deficits are a core feature of neurocognitive disorders, which include delirium, mild cognitive impairment (MCI) and dementia, and represent a decline from previous levels of cognitive function (2). Social cognitive deficits in neurocognitive disorders manifest as difficulties with eye contact, turn taking in conversations, social reciprocation, failure to detect social cues in conversations, and making rude or offensive comments (4). Indeed, the DSM-5-TR now states that for a diagnosis of major neurocognitive disorder, a decline must be observed in one of the cognitive domains, such as memory, language, attention, executive function, perceptual-motor function or *social cognition* (8). For instance, identification of responses requiring interpretation of situational circumstances (i.e., affective theory of mind), but not simple emotions (i.e., emotion perception), is more impaired in people with mild dementia (9).

Early recognition of social cognitive deficits can help identify dementia pathways for individuals, from type [e.g., frontotemporal dementia (FTD) (10)] to progression [e.g., development of behavioral symptoms (11)]. For example, individuals with Alzheimer’s Disease can identify most emotions (e.g., happy, sad, surprise, fear), but have difficulties identifying a range of basic or primary emotions such as disgust or anger in facial expressions, or sarcasm/jokes in conversations (11). There is also evidence for preserved affective empathy in Alzheimer’s Disease and deficits in people with FTD (6) and dementia with Lewy bodies (12). People with Alzheimer’s Disease also show difficulties with identifying some facial emotions (13) and tracking changes in emotions over time (14), There is a lack of research, however, on the differential profiles of social cognitive deficits across the neurocognitive disorders. Routine social cognition assessments can further facilitate appropriate interventions to improve social functioning, with early studies highlighting the potential of psychosocial interventions in strengthening social health for individuals with dementia (14, 15).

Social cognitive skills are needed to maintain social relationships, and vice versa (16). An individual experiencing difficulty reading emotions and/or acting appropriate in social situations may become isolated and lonely. With recent evidence indicating that social isolation is a modifiable risk factor for dementia (17), it is time to consider how we can reliably detect social cognitive deficits in older age and identify changes over time.

Identifying social cognitive deficits continues to be a challenge for multiple reasons. Firstly, current recommendations include a combination of self-report questionnaires, ability based assessments, informant rating, and clinical observation (4), with no standard, or accepted, approach. Secondly, key domains of social cognition (18) typically include broad concepts such as theory of mind, affective empathy, social perception, social behavior (4), and to date, no summary of validated tools has been provided. Furthermore, there are several experimental tasks which are created *ad hoc* or used primarily in research settings to identify social cognitive deficits. Thus, the majority of extant social cognition measures either have not been rigorously developed or psychometrically validated. To our knowledge, there are few questionnaires or neuropsychological tests which assess social cognitive deficits, as most existing brief and comprehensive cognitive assessments omit the social cognition domain (e.g., Mini Mental State Examination).

Research on the development and validation of social cognition assessments, including measurement of social cognitive deficits, in older adults remains limited. We present some of the existing assessments of social cognition developed for use with older adults experiencing neurocognitive disorders. This information can be used to systematically identify social cognitive deficits, understand the social functioning of older adults with neurocognitive disorders and ensure consistency and standardization in the assessment process.

## Overview of social cognition assessments

Thirteen social cognition assessment tools for older adults with neurocognitive disorders (including MCI) are discussed (Table 1). Administration time ranged from 7 min [e.g., Pitfall Intention Explanation task, Pitfall task (12)] to 90 min [e.g., The Awareness of Social Inference Test, TASIT, (24)]. Commonly used assessments were the Interpersonal Reactivity Index (IRI; found in six studies) and TASIT (24) was identified in four studies that assess for social cognition in individuals with neurcognitive disorders. Other scales such as 16-item Toronto Empathy Questionnaire (44) and the 60-item Empathy Quotient (45) have not been used with people with neurocognitive disorders.

**Table 1:** Summary of tools used in studies assessing social cognition in adults with neurocognitive disorders.

Tool name, acronym, and year of initial publication	Type	Subtype	Social cognitive domains	Administration characteristics			Time duration	Psychometric properties		
				No. of items/trials	Intended administration	Tool cost		Validity	Reliability in a sample with neurocognitive disorders	
									Internal consistency	Test–retest
Interpersonal Reactivity Index (IRI) 1983 (9)	Questionnaire	Text	Cognitive and affective empathy	28 Items	Self	Free	Unknown	Discriminant validity in a sample including people with Alzheimer’s Disease: IRI-EC and TASIT-EET: *r* = 0.163, *p* = 0.229 IRI-PT and TASIT-EET: *r* = −0.027, *p* = 0.452 NPI-Ap and TASIT-EET: *r* = −0.037, *p* = 0.433 (19)	–	–
Strange Stories Test (SST) 1994 (20)	Task based		Cognitive and affective theory of mind	16 Items	Interview-administered	Free	Unknown	Discriminant validity in a sample of people diagnosed with amnestic MCI: Raven’s Progressive Color Matrices (*r* = 0.58, *p* = 0.03) (21)	–	–
Reading the Mind in the Eyes Test- revised (RMET) 2001 (22)	Task based	Static, visual	Emotion recognition	36 Items	Interview-administered	Free	10 min	Discriminant validity: RMET scores are worse in Mild Cognitive Impairment 0.52 SD, 95% CI [−0.70, −0.33] and in people with dementia 0.74 SD, 95% CI [−1.13, −0.34] compared to cognitively healthy (23)	–	–
The Awareness of Social Interference Test (TASIT) 2003 (24)	Task based	Dynamic, audiovisual	Emotion recognition, cognitive and affective theory of mind	55 Items	Interview-administered	$250 - $375 for all three parts, including 25 test sheets and 6 response cards	60–90 min	Convergent validity in a sample of people with severe Traumatic Brain Injury: Wechsler Test of Adult Reading (pre-morbid IQ): *r* = 0.26 to 0.50 (25)	–	–
The Awareness of Social Inference Test – short version (TASIT-S) (26)	Task based	Dynamic, audiovisual	Cognitive and affective theory of mind, emotion recognition,	18 Items	Interview-administered	$115 for the test kit, including 25 record forms	15–20 min	Convergent validity in a sample of people with behavioral variant Frontotemporal Dementia and Alzheimer’s Disease: Full version of the TASIT: Part 1 (*r* = 0.897), and Part 2 (*r* = 0.971) (27)	In a sample of people with acquired brain injury: α = 0.96 (26)	In a sample of people with acquired brain injury: *r* = 0.90 (26)
Comprehensive Affect Testing System (CATS) 2006 (28, 29)	Task based	Static: visual items and audio items	Emotion recognition (facial, prosody), social perception (meaning)	298 Trials	Interview-administered	Free for use with an Inquisit Lab license (starting guide $595 for 2 months)	40–50 min for full version	Predictive validity in a cognitively healthy sample: Simple facial scale: β = 0.19 to 0.245 (Step 1 to 4) Complex facial scale: β = 0.422 to 0.336 (Step 1 to 4) Prosody scale: β = 0.206 to 0.013 (Step 1 to 3) Cross-modal scale: β = 0.343 to 0.258 (Step 1 to 3) (30)	–	–
Pitfall intention Explanation Task with Clue Questions (Pitfall task) 2012 (31)	Task based	Static, visual	Cognitive theory of mind (contextual understanding)	1 Scenario with 6 questions	Interview-administered	Unknown	7 min	Convergent validity in samples of people with Alzheimer’s Disease:: MMSE (Pitfall task): *r* = 0.82, *p* < 0.001 MMSE (Cartoons); *r* = 0.50, *p* < 0.001 (32)	In samples of people with Alzheimer’s Disease: α = 0.82 (32)	–
Story-based Empathy test (SET) 2015 (33, 34)	Task based	Static, visual	Cognitive and affective Empathy	18 Trials	Interview-administered	Unknown	15–20 min	Predictive validity: Differentiates between cognitively healthy people and people with behavioral variant of Frontotemporal Dementia and Alzheimer’s Disease (34)	In a sample of people with behavioral variant of Frontotemporal Dementia and Alzheimer’s Disease: α = 0.47 (34)	–
Florida Affect Battery (FAB) 2006 (35)	Task based	Static, visual	Emotion recognition	15 Items	Interview-administered	Unknown	Unknown	Predictive validity: Differentiates between cognitively healthy people and people with frontal or behavioral variant of frontotemporal dementia (36)	–	–
Emotion Recognition Task (ERT) 2007 (37, 38)	Task based	Static, visual	Emotion recognition	16 Items	Computerized	Unknown, can be sourced through Cambridge Cognition	10 min	Predictive validity: differentiates between cognitive healthy people and people with FTD and Alzheimer’s Disease, with ‘anger’ recognition differentiates between the dementia subtypes (39)	–	–
Multifaceted Empathy Test (MET) 2008 (40)	Task based	Static, visual	Cognitive and affective empathy	23 Items	Interview-administered	Unknown	45 min	Predictive validity: Differentes between cognitively healthy people and people with behavioral variant Frontotemporal Dementia (41)	–	–
Edinburgh Social Cognition Test (ESCoT) 2018 (42)	Task based	Dynamic, visual	Cognitive and affective theory of Mind, social perception	11 Subsets: 1 Practice interaction, 5 interactions with social norm violation and 5 interactions without social norm violations.	Interview-administered	Available upon request	30 min	Convergent validity in a cognitively healthy sample including older adults: RME: *r* = 0.33, *p* < 0.01 SNQ: *r* = 0.19, *p* < 0.05 (42)	–	–
The brief Assessment of Social Skills- Dementia (BASS-D) 2020 (43)	Task based	Static, visual	Emotion recognition, face identification, empathy, theory of mind, social inhibition, social reasoning, memory for faces	109 Items	Interview-administered	Available upon request	30–40 min for full version	Convergent validity in a sample of people with dementia (type not specified): TASIT EET: *r* = 0.806, *p* < 0.1 BEES: *r* = 0.367, *p* < 0.05 TASIT SI-E: *r* = 0.32, *p* < 0.05 (43)	In a sample of people with dementia (type not specified): α = 0.80 (43)	–

Social cognition assessments mostly targeted ability-based only approaches [*n* = 10/13, Strange Stories Task (SST) (20), TASIT (24), TASIT-S (26), Comprehensive Affect Testing System (CATS) (28), Story-based Empathy Test (SET) (33, 34), Florida Affect Battery (FAB) (35), Emotion Recognition Task (ERT) (37, 38), Multifaceted Empathy Test (MET) (40), Edinburgh Social Cognition Test (ESCoT) (42), and Brief Assessment of Social Skills – Dementia (BASS-D) (43)]. Questionnaire-based [Interpersonal Reactivity Index (IRI) (9)] and mixed approaches [PIE (31)] were less frequent.

### Assessments over time

Several social cognition assessments were originally developed for general purposes or in other populations, but have since been adapted for or validated with people with dementia. For instance, the IRI is widely used to assess empathy in the general population (46), and was later validated in the context of dementia (9) to better cover empathic concern and personal distress features (47). Similarly, the TASIT was originally developed to assess social cognitive skills following a traumatic brain injury (48), and was later validated for people with dementia (49).

However, other measures lacked specific development with individuals with dementia [e.g., CATS (28, 29), Pitfall Task (31), SET (13, 14)], yet they contain multiple subtests aimed at assessing various aspects of social cognition (e.g., emotion recognition, intention attribution and causal inference).

The majority of instruments focused on one or two domains, predominantly on emotion recognition (identifying a facial expression) or cognitive or affective theory of mind (taking others’ perspectives). Emotion recognition (TASIT, CATS, FAB, ERT) and cognitive or affective theory of mind (TASIT-S, Pitfall Task, ESCoT) were most common, followed by either cognitive and/or affective empathy (IRI, SET, MET).

**Figure 1:**
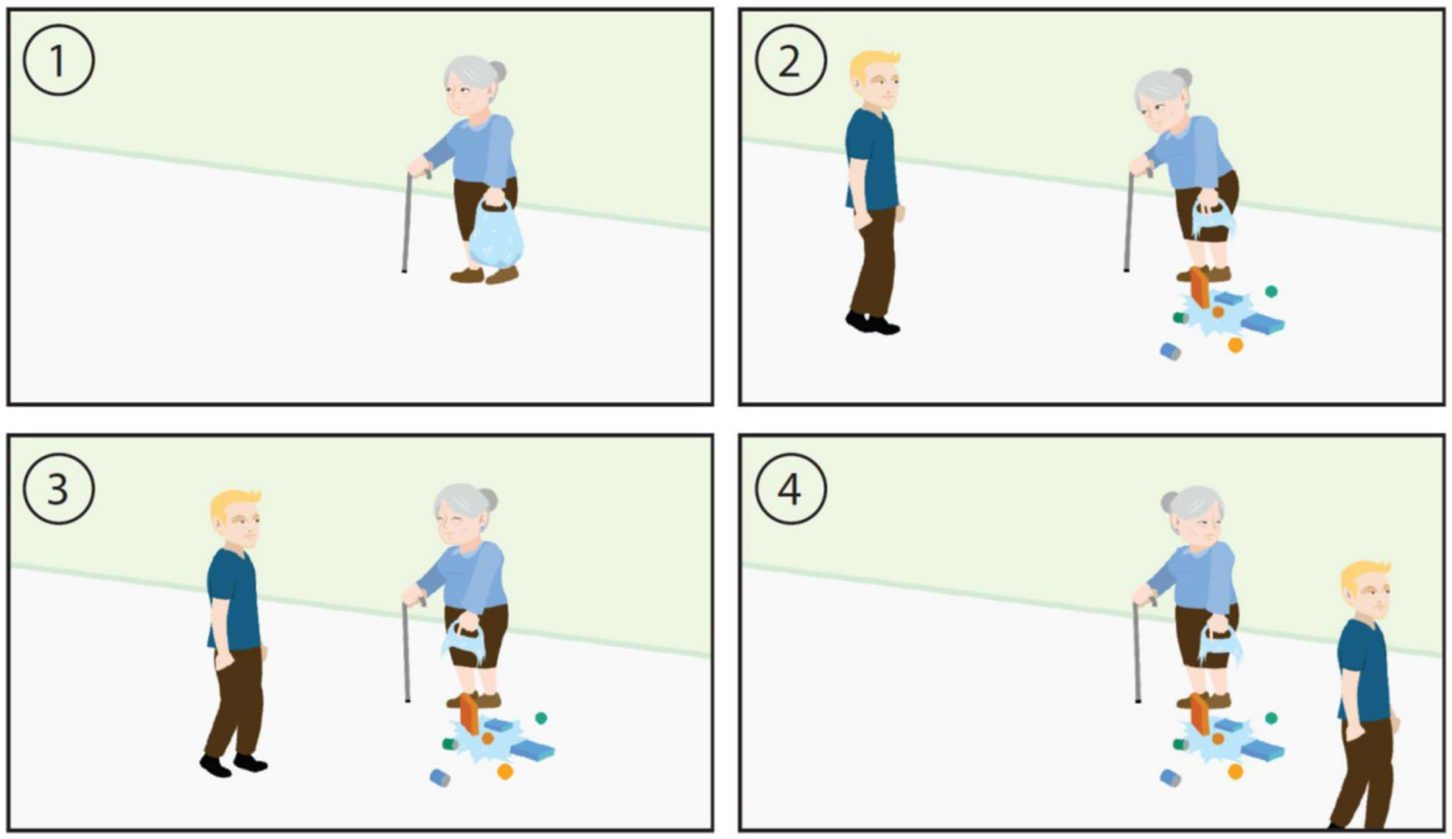
Scenario from the Edinburgh Social Cognition Test (ESCoT).

More recently developed assessments such as the ESCoT (42) in 2018 and BASS-D (43) in 2020 cover more detailed social cognition components (e.g., not just a singular domain such as cognitive or affective theory of mind) in a more ecological valid manner (Figure 1). For instance, the ESCoT involves presenting animations of social interactions and the individual is asked about the thoughts and feelings of the characters and the norms that apply in each scenario (Figure 1). One scenario depicts a young man walking past an older woman who’s shopping bag has ripped, causing the contents to fall onto the street. The interviewer asks what the older woman thought and felt at the start of the scenario and at the end (when the young man keeps walking instead of stopping to help her) and what the social norm would be in the scenario (e.g., to help the woman).

Similarly, the BASS-D (43) presents a comprehensive set of photos of faces and videos of interactions to assess the individual’s ability to recognize emotions, identify facial features and expressions, provide appropriate empathic responses, and exhibit social inhibition and reasoning.

### Psychometric properties

To the best of our knowledge, all assessments have been tested for validity and some for reliability (6/13 46%) within samples including people with neurocognitive disorders (most commonly reported FTD). Most reported internal consistency rather than test–retest (1/13; 7%; Table 1). Internal consistency ranged from low to moderate (e.g., SET; α = 0.47) to high (TASIT-S; α = 0.96). Validity ranged from low (e.g., CATS) to high (e.g., FAB), with most measures exhibiting moderate to high convergent validity. We were unable to find instruments that were tested for responsiveness to interventions. All of the above mentioned tests have been validated with people with neurocognitive disorders: IRI (50), SST (20), TASIT (19), TASIT-S (27), CATS (51), Pitfall Task (31), SET (34), FAB (52), ERT (37, 38), MET (40), ESCoT (53), and BASS-D (43).

## Future directions

Indexing a given social cognitive domain is challenging, due to the current lack of consensus on the definition and theoretical derivation of cognitive domains in neurocognitive disorders. Thus, several assessments provided considerable conceptual overlap between domains. The listed measures assessed multiple domains of social cognition, but tended to focus primarily on the understanding of social situations. This can be problematic as questionnaires (e.g., IRI) often have the limitations of self-report measures, requiring individuals to have insight into their own strengths and weaknesses, which may not be possible during later stages of dementia.

While social reasoning, identifying and remembering faces, and social disinhibition add depth to the assessment of social cognition, a key component remains to be assessed: namely, *social behavior*. This domain was largely missing and includes skills such as eye contact, turn taking, asking open ended questions, using humor, understanding puns and jokes, and keeping conversations going. Given the ability of emotion recognition and empathy tasks to differentiate between Frontotemporal Dementia and Alzheimer’s Disease (34), it is plausible that individuals who show deficits in these skills may also have difficulties with responding appropriately in social situations. These concepts are understandably difficult to self-report. The main issue is that tools developed to date rely on interpreting the thoughts and feelings of an actor or character, thereby limiting the ecological validity of the tests. The use of actors or characters adds a layer of “pretending” to the tests, which it could be argued require a certain level of theory of mind. The current tests also involve selecting ‘appropriate’ responses based on arbitrary criteria imposed by the researcher, rather than real world dynamic interactions (54). Observational tests might be more suitable to assess social skills (e.g., turn taking and keeping conversations going). However, observational measurements are traditionally time-consuming (e.g., ethnographic studies) and are difficult to standardize, so harnessing the power of technology might be a suitable approach.

The integration of technology in the measurement of social cognition has the potential to transform our understanding of the human brain and behavior. Virtual reality could assess a range of complex social cognitive processes, ranging from emotion recognition, social perception, and cognitive or affective theory of mind, in a more ecologically valid way than traditional approaches (55). Virtual reality can further manipulate social cues, such as facial expressions and tone of voice, capturing the nuance of social interactions and cognitive processes in a controlled, yet naturalistic environment. This method can provide valuable insights into the underlying mechanisms of social cognition, create highly customized social situations tailored to the individual’s specific social cognitive deficits, enabling targeted interventions and treatments.

Technological advances in automatic tracking capabilities (e.g., eye, microfacial expressions) have the potential to further deepen our understanding, and thus definition of, appropriate and inappropriate social behaviors and reactions in individuals with neurocognitive deficits (56). Whilst detection and analysis of common and basic expressions such as happiness, sadness, surprise, anger, disgust has been achieved, recent advancements in machine learning and artificial intelligence have led to the development of more sophisticated algorithms that can detect even more subtle facial movements. These algorithms can now identify patterns in facial expressions that has real-world applications and responses, such as nervousness, disagreement and contempt (57), enabling more accurate detection of microfacial expressions associated with other emotions.

Other common forms of technology such as wearable devices, which can collect physiological data including heart rate variability and skin conductance, can be used to implore the impact of social cognitive processes on the autonomic nervous system, providing a more nuanced understanding of the physiological processes involved in social cognition (58). As technology continues to evolve, we can expect increasingly sophisticated and accurate assessments of social cognition, which will support our understanding and eventual interventions and treatments for individuals with social cognitive deficits.

### Recommendations

The use of valid and standardized outcome measures for the assessment of social cognition in older adults with major neurocognitive disorders is necessary to support early intervention and treatment of dementia conditions, as well as strengthening epidemiological studies to further enable our understanding of the trajectory of social cognitive deficits and its association with other factors.

Here we propose four key recommendations to aid the establishment of comprehensive social cognition assessments for older adults with major neurocognitive disorders.

#### Tool selection and administration

In the absence of other tools, we recommend multicomponent assessments of social cognition, such as the BASS-D and ESCoT, to help increase identification and recognition of social cognitive performance. However, whilst considering which aspects of social cognition should be included in a battery for examining older adults with neurocognitive disorders, a holistic approach should be adopted. While theory of mind is undoubtedly a critical aspect, other components such as emotion recognition, empathy, social perception, and social behavior should also be considered during clinical appraisals. These components collectively provide a more nuanced assessment of social cognitive abilities, allowing for more accuracy of deficits and the development of targeted interventions. However, the selection and delivery of social cognition assessment depends on several factors, including the research objectives, the specific population under study, psychometric properties, cultural appropriateness, the clinical utility of these tools and available resources. Embedding social cognition batteries into standard practice, especially when facing time and funding limitations often present in clinical settings, requires a strategic and pragmatic approach. Practical steps to consider are prioritizing key domains (e.g., focus on a subset of domains most indicative of an individual’s overall social cognitive functioning to streamline the assessment process), using brief and targeted assessment tools that provide meaningful insights within a shorter timeframe, integrating assessments with existing tools, providing flexible administration and leverage technology to automate scoring, analyses and feedback. At times, the implementation of social cognition assessment may need to be selectively based on clinical judgement and determine which individuals are most likely to benefit from a social cognition assessment, considering their presenting symptoms, history, and treatment goals.

#### Enable better, and more accurate diagnostic measures

We need screening tools for use in research and clinical settings to complement existing cognitive assessments, which often exclude the domain of social cognition. These new measures should be rigorously tested, validated, appropriate, and multidimensional. Social cognitive measures used with other populations (e.g., traumatic brain injury, healthy older adults) could possibly be further validated with people with MCI or dementia [e.g., Emotion Recognition Task (59)] to further identify how affective and cognitive theory of mind are supported by different brain functionalities. There is a lack of knowledge on the timeframe required to detect clinically significant changes and the feasibility of using alternative, targeted versions for assessments carried out within time-limited interventions. Such adaptations may be particularly valuable in clinical settings where efficiency and brevity are necessary considerations. Furthermore, while emerging research suggests the potential utility of social cognition measures in indexing treatment effects more broadly (59), empirical investigations are needed to establish their validity and clinical significance, particularly for individuals with neurocognitive disorders. Currently, there is limited understanding of how quickly treatment interventions may lead to observable improvements in social cognition. Well-designed studies that track individuals over time can provide insights into the temporal dynamics of treatment effects, helping establish realistic expectations for intervention outcomes (60).

#### Explore the potential additive value of comprehensive social cognitive evaluation

Studies should investigate how social cognition tools can enhance the detection, characterization, and monitoring of cognitive changes in individuals with various forms of dementia. Social cognition measures were predominantly concentrated on individuals with FTD, although there is growing interest in exploring their broader applicability across other neurodegenerative conditions, including MCI and various forms of dementia. Indeed, social cognition tools hold promise beyond FTD and may offer additional insights into the cognitive profiles of individuals with MCI and dementia (60, 61). Future research needs to establish the potential additive value of social cognition measures to traditional cognitive assessments, and the practical implications and clinical utility of incorporating social cognition tools into cognitive assessments on diagnostic accuracy, treatment planning, and prognosis. We expect that specific dimensions will be affected by different (or different rates of progress of) neurocognitive disorders. Multidimensional measures may aid in differential diagnoses and identify potential social cognitive domains requiring interventions or support.

#### Harness digital technologies to support ecological measures

Developing more sensitive tools using digital technology (such as virtual reality, eye tracking, real-time AI assisted scoring of social behavior) would allow more accurate and sensitive measurement of social cognition, both at home and in the clinic. These tools are likely to be more ecologically valid and engaging assessments, which can enhance the sensitivity of detecting social cognitive impairments across different clinical populations. Technology might combine both performance-based measures and subjective self-report measures to capture subtle, multidimensional aspects of social cognition decline. Adaptive testing approaches, for example, can tailor the assessment difficulty to the individual’s cognitive abilities, capturing the entire spectrum of social cognitive deficits.

## Conclusion

Moving forward, more work is needed to elucidate the effective measurement of social cognition and to develop a multidimensional and more ecologically valid diagnostic social cognitive measures that have utility in the clinic. We encourage the development and refinement of existing tools to cover more broadly social behavior to enable more accurate depiction of one’s social functioning. This can be supplemented with technology to enable routine assessments in the clinic.

## Data availability statement

The original contributions presented in the study are included in the article/supplementary material, further inquiries can be directed to the corresponding author.

## Author contributions

SS and JS: conceptualization, methodology, supervision, writing – original draft, and writing – review and editing. AM: article search and synthesis and writing – review and editing. All authors contributed to the article and approved the submitted version.

## Funding

SS declares payments for lectures from NYU Sydney and University of Sydney, and grant funding from Dementia Australia Research Foundation (not for current manuscript) and grant funding from EU-JPND and NHMRC Australia (not for current manuscript). JS receives a Research Theme Fellowship from Western Sydney University.

## Conflict of interest

The authors declare that the research was conducted in the absence of any commercial or financial relationships that could be construed as a potential conflict of interest.

## Publisher’s note

All claims expressed in this article are solely those of the authors and do not necessarily represent those of their affiliated organizations, or those of the publisher, the editors and the reviewers. Any product that may be evaluated in this article, or claim that may be made by its manufacturer, is not guaranteed or endorsed by the publisher.
